# Perceptions of hospital electronic health record (EHR) training, support, and patient safety by staff position and tenure

**DOI:** 10.1186/s12913-024-11322-3

**Published:** 2024-08-20

**Authors:** Joanne Campione, Helen Liu

**Affiliations:** 1 Westat, Durham, North Carolina US; 2 Westat, Rockville, Maryland US

**Keywords:** Patient safety, Health IT, Electronic health records (EHR), Hospital EHR system

## Abstract

**Background:**

Hospitals rely on their electronic health record (EHR) systems to assist with the provision of safe, high quality, and efficient health care. However, EHR systems have been found to disrupt clinical workflows and may lead to unintended consequences associated with patient safety and health care professionals’ perceptions of and burden with EHR usability and interoperability. This study sought to explore the differences in staff perceptions of the usability and safety of their hospital EHR system by staff position and tenure.

**Methods:**

We used data from the AHRQ Surveys on Patient Safety Culture^®^ (SOPS^®^) Hospital Survey Version 1.0 Database and the SOPS Health Information Technology Patient Safety Supplemental Items (“Health IT item set”) collected from 44 hospitals and 8,880 staff in 2017. We used regression modeling to examine perceptions of EHR system training, EHR support & communication, EHR-related workflow, satisfaction with the EHR system, and the frequency of EHR-related patient safety and quality issues by staff position and tenure, while controlling for hospital ownership type and bed-size.

**Results:**

In comparison to RNs, pharmacists had significantly lower (unfavorable) scores for EHR system training (regression coefficient = -0.07; *p* = 0.047), and physicians, hospital management, and the IT staff were significantly more likely to report high frequency of inaccurate EHR information (ORs = 2.03, 1.34, 1.72, respectively). Compared to staff with 11 or more years of hospital tenure, new staff (less than 1 year at the hospital) had significantly lower scores for EHR system training, but higher scores for EHR support & communication (*p* < 0.0001). Dissatisfaction of the EHR system was highest among physicians and among staff with 11 or more years tenure at the hospital.

**Conclusions:**

There were significant differences in the Health IT item set’s results across staff positions and hospital tenure. Hospitals can implement the SOPS Health IT Patient Safety Supplemental Items as a valuable tool for identifying incongruity in the perceptions of EHR usability and satisfaction across staff groups to inform targeted investment in EHR system training and support.

**Supplementary Information:**

The online version contains supplementary material available at 10.1186/s12913-024-11322-3.

## Background

As a key component of digital healthcare, electronic health records (EHR) have the potential to improve the quality, efficiency, and coordination of patient health care services [1, 2]. Usability concerns and unintended negative consequences of EHR adoption, however, can negatively affect quality of care and patient safety [3–5]. Moreover, studies have found that EHRs can potentially cause technology-induced errors and disruptions in clinical workflows due to poor design, training, usability, implementation, or support [6, 7].

One of the leading reasons for poor outcomes following EHR adoption may be the lack of consideration for how the system will interface with end-users and the existing organizational structure [8]. Thus, it is important for a hospital to adequately train all staff members in using an organization’s EHR system and to encourage feedback from staff about the safety and usability of the system [9]. Nurses, pharmacists, physicians, technicians and other clinical staff are end-users that are well-suited to provide input about the EHR usability, the EHR system’s impact on clinical workflow, and problem resolution. Due to their first-hand experience and interaction with EHRs during patient care, these clinical staff can identify when patients are at risk for an EHR-based complication, error, or adverse event and should be called upon to provide both feedback and suggestions for improving the usability and safety of EHRs.

Patient safety culture is a core component of a high reliability organization and often viewed as a contextual factor that shapes staff behaviors, perceptions, attitudes, and commitment and improves care quality [10–23]. Studies have found that management have more positive perceptions of safety culture in comparison to frontline clinical staff [15, 24, 25]. An approach for organizations to measure, understand, and improve patient safety is to survey their staff about their perception of the culture of patient safety in the organization. One commonly used survey is the Agency for Healthcare Research and Quality (AHRQ) Surveys on Patient Safety Culture^®^ (SOPS^®^) Hospital Survey.

The safety performance of many hospital EHR systems continues to be unreliable or unknown [26]. Hospitals have been strongly encouraged to measure and address EHR-related patient safety to improve EHR usability, interoperability, and safety [27–29]. In 2017, to supplement the SOPS Hospital Survey, AHRQ developed the SOPS Health Information Technology Supplemental Item Set for hospitals (“Health IT item set”) to help hospitals assess how the use of the EHR system affects patient safety from the perspectives of providers and staff working in their facility [30].

### Study objective

The objective of this study was to examine the results of the 2017 pilot study of the Hospital SOPS Health IT item set and to further examine the results by staff position and hospital tenure. The objective of the study is to provide new information which indicates a need for targeted training programs or additional support strategies and resources to certain staff types within an organization. To our knowledge, there are no studies to date that examine differences in the perception of EHR safety and usability by hospital tenure (e.g. < 1 year at hospital, 1–5 years, 6–10 years, > 10 years). Examining these differences across tenure categories introduces a novel comparison between new users and those who are more accustomed to the system. Furthermore, examining perceptions of EHR safety and usability based on hospital tenure can reveal if longer-term users are adapting to the system over time or if there are persistent issues leading to dissatisfaction.

## Methods

### Data source

We used cross-sectional, individual staff responses to the AHRQ SOPS Hospital Survey Version 1.0 and the Health IT items from 44 hospitals that voluntarily participated in the 2017 pilot study of the Health IT item set [31, 32]. Survey questions from the psychometrically-sound Health IT item set are shown in Supplement Table S1 in the order in which they appeared in the survey [30].

### Study sample

The survey results from the 44 hospitals included responses from 9,351 individual staff that took the Hospital SOPS Version 1.0 with the Health IT item set who use their hospital’s EHR system to enter or review patient information. We excluded staff with a non-response (missing) or non-substantive response (i.e., does not apply and don’t know) to tenure at the hospital (*n* = 243) or staff position (*n* = 63). We also removed individuals who passed the filter item, but still had all missing responses to the Health IT items (*n* = 97). Lastly, we excluded staff (*n* = 68) who did not complete the core survey. The final study sample resulted in 8,880 staff members from 44 hospitals with an average of 200 staff respondents per hospital (range: 5 staff – 402 staff). Staff eligible to take the survey included permanent staff and contract staff who consistently work in the hospital. More information about the 2017 Pilot Study response rates, hospital demographics, and results can be found on AHRQ’s website [33].

### Dependent measures

The Health IT item set included the following 11 measures: (a) five items on the frequency of EHR patient safety and quality issues; (b) a three-item composite measure about EHR system training; (c) three items on EHR workflow and work process (“Workflow”); (d) a three-item composite measure about EHR system support & communication and (e) one item on the overall satisfaction with the EHR system. For the frequency of issues questions, we created “high frequency” dichotomized variables from these questions representing responses of 6 or more times in the past 3 months. For the composite measures, we calculated mean scores of the constituent items based on a 5-point scale (e.g., 1 = strongly disagree; 2 = disagree; 3 = neither agree nor disagree, 4 = agree, 5 = strongly agree) where higher ratings are more favorable. The individual workflow items have the same response options as the composite measures. Lastly, we created a dichotomized variable representing when a person was dissatisfied or very dissatisfied with the EHR system. Supplement Table S1 provides more detail about the Health IT item text, response options, and scoring of the items and measures.

### Staffing variables

We used responses to *“What is your staff position in this hospital?”* from the Hospital SOPS to make 7 categories of self-reported staff position: (1) Administration, Management (“management”), (2) Information Technology (“IT staff”), (3) Medical Assistant, Other Clinical (“other clinical”), (4) Non-Clinical, Office, Social Work, Case Management (“non-clinical”), (5) Pharmacist, Technician (“pharmacists”), (6) Physician, Surgeon, Resident (“physicians”), and (7) Registered Nurse (“RNs”). Nurses without an RN degree fall under “other clinical”. Supplement Table S2 provides more detail about position groups.

We used responses to *“How long have you worked in this hospital?”* from the Hospital SOPS to make four categories of hospital tenure: (1) Less than 1 year, (2) 1 to 5 years, (3) 6 to 10 years, and (4) 11 or more years. The analysis of the Health IT measures by hospital tenure excluded the IT staff (*n* = 332) because these individuals were most likely the staff who provided the EHR training and support, thus offering a potential bias in perceptions of effectiveness and adequacy. The sample for the tenure analysis was 8,548 staff.

### Analysis

We ran analysis of variance (ANOVA) using the Tukey-Kramer post-hoc test to look for significant differences in the mean scores and frequencies (i.e., variability) across staff position and hospital tenure for the 11 EHR measures. Tukey-Kramer tests all possible pairs of all groups for unequal sample sizes while controlling for experiment-wise error rate [34].

We used multivariable regression models to see if differences found with ANOVA remained significant after controlling for ownership type and bed-size category. We controlled for ownership type and bed-size category because these hospital-level factors have been shown to be associated with Hospital SOPS measures [35] and may also be associated with the perceptions of usability and safety of a hospital’s EHR system. For the models with continuous dependent variables (workflow items and composite measures), we used a mixed effects regression model that included the use of a hospital ID to address nesting within the 44 hospitals. For the dichotomized measures, we used a logistic regression model. Due to the lack of variability associated with a binary outcome and the reduction in the model’s degrees of freedom when the 44 hospital IDs are included, we excluded hospital ID from the logistic regression models. However, the logistic regression models still controlled for hospital ownership type and hospital bed-size category. For the regression models with staff position as the key independent variable, we used RNs as the reference group, because this group was the most prevalent staff position among the respondents. For the regression models with hospital tenure as the key independent variable on an ordinal scale, we used the largest of the two extreme categories, 11 or more years, as the reference group. All analyses were conducted using SAS 9.4. The pilot test of the Health IT item set was IRB approved. The data received by the authors had de-identified hospital and staff.

## Results

### Outcome scores and frequencies

The aggregate results of all 11 EHR measures and the number of valid responses per measure are shown in Table 1. Valid responses exclude missing responses (i.e., item left blank) and non-substantive responses such as “does not apply” or “don’t know”. All of the items had 80% or higher valid responses. The EHR issues of “Important information was hard to find” was reported to occur at a high frequency by 28% of the respondents, followed by “Information was not complete” by 25% of the respondents. The EHR system training composite measure had a statistically significantly higher, more favorable, mean score than the EHR support & communication composite measure (3.53 vs. 3.29; *p* < 0.0001). Lastly, 20% of the staff reported dissatisfaction with the EHR system.

**Table 1:** Health IT outcome measures mean percentages and scores (*N* = 8880 staff)

Measure		# of Valid Responses^c^
*Composite Measures*	**Mean (SD)**	
EHR system training (composite measure)	3.53 (0.90)	8146
EHR support & communication (composite measure)	3.29 (0.84)	7720
*Quality Issues* ^*a*^	**Percent (SD)**	
Information was not complete	25.2% (0.43)	7473
Information was not accurate	19.5% (0.37)	7521
Important information was hard to find	28.0% (0.45)	7751
Information was entered into the wrong patient record	3.4% (0.18)	7493
Incorrect information was copied and pasted	11.0% (0.31)	7170
Dissatisfied or very dissatisfied with hospital’s EHR system	20.2% (0.40)	8808
*Workflow*	**Mean (SD)**	
There are enough EHR workstations available	3.66 (1.11)	8272
NOT required to enter the same info in too many places^b^	2.55 (1.09)	7933
There are NOT too many alerts or flags in our EHR system^b^	3.17 (0.94)	7705

Note: For the Composite Measures and Workflow items, the range is 1–5 and a higher mean is better. For the Quality Issues, lower percent is better. SD = standard deviation

^a^ Percent “high frequency” – issue happened 6 or more times in the past 3 months

^b^ Change in measure description from the original question to align with reverse-scoring

^c^ Valid responses exclude missing (i.e., non-response) and non-substantive responses. All of the items had, on average, 80% or higher valid responses. Non-valid responses for Information not accurate ranged from 11% among physicians to 22% pharmacists; Non-valid responses for Dissatisfaction of EHR system was less than 2% for all staff positions; Non-valid responses for EHR support & communication ranged from 12% among tenure 6–10 years to 15% among tenure < 1 year; Non-valid responses for EHR training ranged from 8% among tenure 11 or more years to 10% among tenure < 1 year

The distribution of the 44 hospitals by ownership type was: Nongovernment not-for-profit (84%), Government-owned (9%), and For-profit (7%), and the distribution by bed-size category was: 100–299 beds (46%), 50–99 beds (36%), and 300 or more beds (18%).

As shown in Table 2, the distribution of the staff position groups was: RNs (41.9%), other clinical (17.9%), non-clinical (13.0%), pharmacists (10.9%), management (6.7%), physicians (5.9%), and IT staff (3.7%). The distribution of hospital tenure was: 1 to 5 years (37.2%), 11 or more years (34.7%), 6 to 10 years (18.0%), and less than 1 year (10.2%).

**Table 2:** Survey results of key outcome measures by staff position and hospital tenure

	EHR System Composite Mean Score		High Frequency^a^ of a Patient Safety Quality Issue (% among the group)					Dissatisfaction (% among group)	Workflow Mean Score		
	Training	Support and Communication	Info not complete	Info not accurate	Important Info hard to find	Info entered into wrong patient	Incorrect info copied and pasted	Dissatisfied with EHR System	Enough workstations	Don’t have to re-enter info	Not too many alerts
**Position (% of 8880)**											
Management (6.7%)	3.60	3.37	32.2%	23.8%	40.3%	4.2%	16.5%	27.3%	4.01	2.52	3.27
IT staff (3.7%)	3.68	3.50	36.6%	28.5%	29.1%	13.8%	12.0%	17.7%	4.03	2.98	3.17
Other clinical (17.9%)	3.56	3.35	19.2%	15.9%	23.4%	2.9%	10.0%	17.5%	3.62	2.71	3.27
Non-clinical (13.0%)	3.54	3.38	18.5%	16.6%	15.8%	3.3%	5.0%	12.2%	3.83	2.84	3.30
Pharmacists (10.9%)	3.43	3.20	23.4%	17.6%	23.7%	2.3%	5.2%	14.9%	3.72	2.87	3.12
Physicians (5.9%)	3.49	3.27	38.5%	32.8%	45.0%	4.5%	23.8%	32.1%	3.57	2.51	2.77
RNs (41.9%)	3.51	3.25	26.1%	19.0%	29.8%	2.8%	11.6%	22.6%	3.55	2.31	3.15
**Hospital Tenure (% of 8548)** ^**b**^											
Less than 1 year (10.2%)	3.43	3.40	19.4%	15.1%	25.6%	2.7%	6.7%	19.5%	3.73	2.64	3.29
1 to 5 years (37.2%)	3.48	3.30	23.5%	19.5%	27.2%	3.1%	10.6%	18.6%	3.66	2.59	3.22
6 to 10 years (18.0%)	3.56	3.28	25.9%	19.0%	28.3%	3.6%	13.4%	19.8%	3.58	2.48	3.07
11 or more years (34.7%)	3.56	3.24	27.3%	20.2%	29.3%	2.8%	11.5%	22.7%	3.66	2.48	3.12

Note: For the Composite Measure and Workflow items, the range is 1–5 and a higher mean is better. For the Quality Issue and Dissatisfaction items, lower percent is better

^a^ “High Frequency” – staff member reported discovery of the issue 6 or more times in the past 3 months

^b^ The Information Technology staff (*n* = 332) were excluded from tenure analysis

**Fig. 1:**
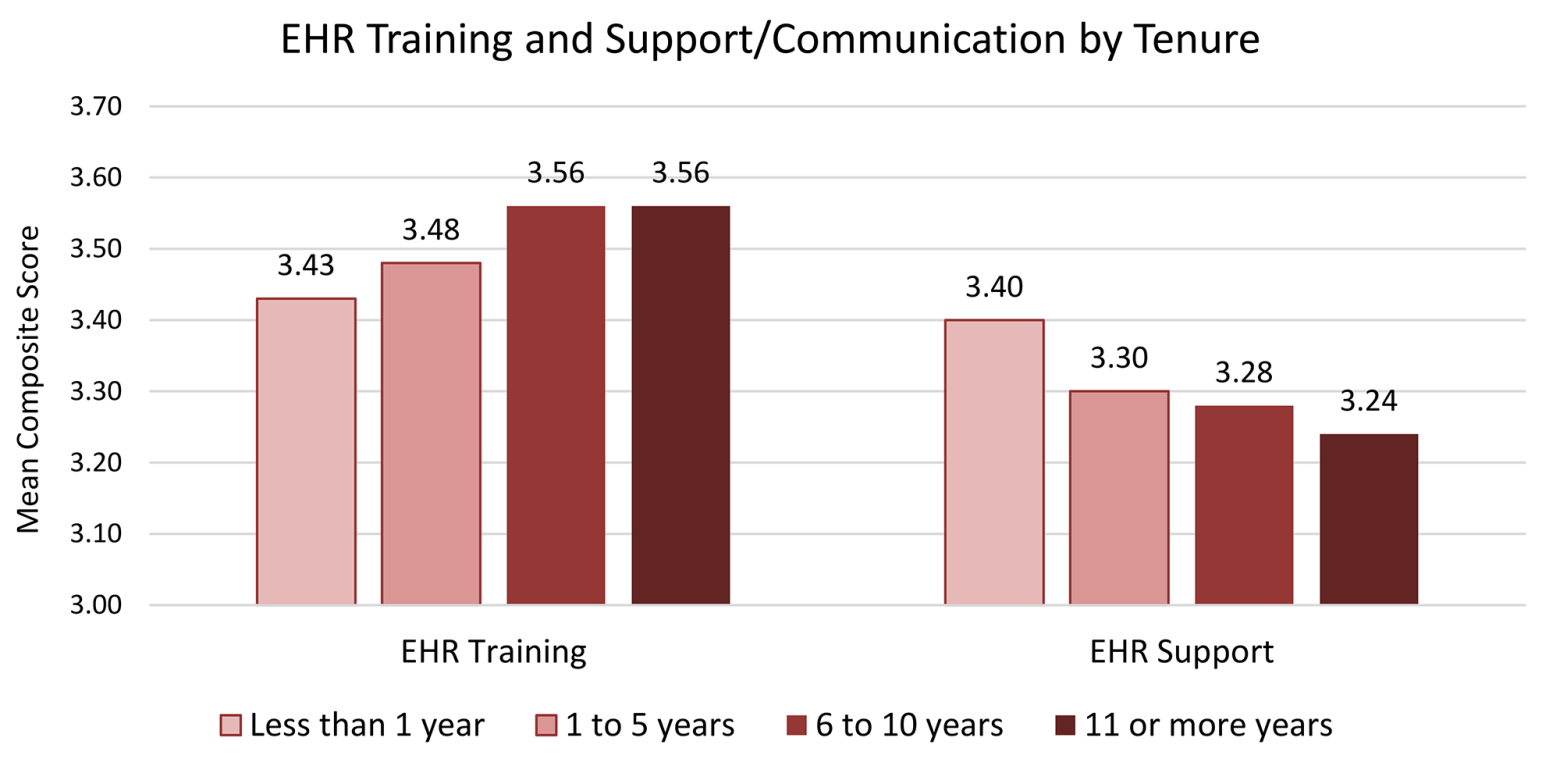
Mean Scores of the EHR System Training and Support by Hospital Tenure Note: Hospital tenure included 8548 staff. The Information Technology staff (*n* = 332) were excluded from tenure analysis

Table 2 shows the results for the 11 outcome measures by staff position and hospital tenure. The mean score of the EHR system training composite measure was highest among IT staff (3.68) and tenure of 6 to 10 and 11 or more years (3.56) but was lowest for pharmacists (3.43) and tenure less than 1 year (3.43). The mean score of the EHR support & communication composite measure was highest for the IT staff (3.50) and tenure less than 1 year (3.40), while it was lowest for pharmacists (3.20) and tenure of 11 or more years (3.24). Figure 1 shows that there is an increase in the mean value for EHR training by tenure and a decrease in the mean value of EHR support & communication by tenure. High frequency of “Information was not accurate” was highest among physicians (32.8%) and staff with tenure of 11 or more years (20.2%) while lowest among other clinical (15.9%) and tenure of less than 1 year (15.1%). Dissatisfaction with the EHR system was highest among physicians (32.1%) and tenure of 11 or more years (22.7%) and lowest among non-clinical (12.2%) and tenure of 1 to 5 years (18.6%).

### Regression results: staff position

The results of each regression model for the 11 measures can be found in Tables 3 and 4 and Supplement Tables S3–S6. In Table 3, we show the multivariable regression results by staff position for the two composite measures, one quality issue, and dissatisfaction with EHR system. For the training composite, the pharmacists had significantly less favorable mean scores (β = -0.07; *p* = 0.0469) compared to RNs. For the support & communication composite, the RNs had significantly less favorable scores than IT staff, non-clinical, management, and other clinical (*p* < 0.001 for all four). For both of the composite measures, the coefficient estimates for physicians were not significantly different from the scores of RNs.

**Table 3:** Regression results: EHR training, support, inaccuracy, and dissatisfaction by staff position

Independent Variables	EHR System Training		EHR System Support and Communication		High Frequency of Information was Not Accurate		Dissatisfied with EHR	
	Coef Estimate	Pr > |t|	Coef Estimate	Pr > |t|	Odds Ratio	95% CI	Odds Ratio	95% CI
Staff Position (reference = RN)								
Administration, management	0.10*	0.0139	0.14**	0.0002	1.34*	(1.07,1.68)	1.27*	(1.04,1.55)
Information technology	0.21**	0.0001	0.28**	< 0.0001	1.72*	(1.29,2.28)	0.69*	(0.51,0.92)
Other clinical	0.06*	0.0394	0.11**	< 0.0001	0.81*	(0.68,0.96)	0.73*	(0.62,0.85)
Non-clinical, office, social work	0.04	0.2570	0.15**	< 0.0001	0.85	(0.70,1.04)	0.47*	(0.39,0.57)
Pharmacist, technician	-0.07*	0.0469	-0.03	0.3357	0.91	(0.74,1.13)	0.61*	(0.50,0.74)
Physician, resident	-0.03	0.4652	0.02	0.5964	2.03*	(1.64,2.52)	1.69*	(1.38,2.07)
Bed size (reference = 100–299)								
50–99	0.40**	0.0097	0.17	0.2188	0.93	(0.77,1.12)	1.16	(0.99,1.36)
300 or more	-0.36**	< 0.0001	-0.28**	< 0.0001	1.08	(0.94,1.23)	0.95	(0.84,1.07)
Ownership (reference = Nongovt not-for-profit)								
Government non-federal	-0.53**	0.0029	-0.44**	0.0066	0.96	(0.77,1.21)	2.28*	(1.90,2.73)
For-profit	-0.57**	0.0028	-0.47**	0.0062	0.85	(0.58,1.23)	0.64*	(0.45,0.91)

Note: *n* = 8880; positive regression coefficient indicates better score on the mean composite measure;

odds ratio > 1 (unfavorable) indicates greater likelihood of high frequency inaccuracy and EHR system dissatisfaction

Staff position estimates are in comparison to RNs.

RN = Registered Nurse; Nongovt = nongovernment; Coef = coefficient; Pr = probability; CI = confidence interval

* significant at *p* < 0.05

** significant at *p* < 0.01

**Table 4:** Regression results: EHR training, support, inaccuracy, and dissatisfaction by Hospital Tenure

Independent Variables	EHR Training		EHR Support & Communication		High Frequency of Information was Not Accurate		Dissatisfied with EHR	
	Coef Estimate	Pr > |t|	Coef Estimate	Pr > |t|	Odds Ratio	95% CI	Odds Ratio	95% CI
Hospital Tenure (reference = 11 or more years)								
Less than 1 year	-0.15**	< 0.0001	0.16**	< 0.0001	0.70*	(0.56,0.88)	0.83	(0.69,1.01)
1 to 5 years	-0.11**	< 0.0001	0.05*	0.0387	0.96	(0.84,1.10)	0.79*	(0.70,0.90)
6 to 10 years	-0.01	0.6160	0.02	0.4285	0.93	(0.78,1.10)	0.85*	(0.73,0.99)
Bed size (reference = 100–299)								
50–99	0.35	0.0629	0.16	0.2464	0.90	(0.74,1.09)	1.19*	(1.01,1.40)
300 or more	-0.38**	0.0035	-0.30**	< 0.0001	1.13	(0.99,1.29)	1.00	(0.88,1.13)
Ownership (reference = Nongovt not-for-profit)								
Government non-federal	-0.57**	0.0061	-0.49**	0.0032	0.92	(0.73,1,17)	2.18*	(1.81,2.62)
For-profit	-0.50*	0.0213	-0.53**	0.0025	0.85	(0.58,1.25)	0.62*	(0.43,0.89)

Note: *n* = 8548 (excluding Information Technology staff); a positive regression coefficient indicates better score on the mean composite measure;

odds ratio > 1 (unfavorable) indicates greater likelihood of high frequency inaccuracy and EHR system dissatisfaction

Tenure estimates are in comparison to staff who worked in the hospital 11 or more years

Nongovt = nongovernment; Coef = coefficient; Pr = probability; CI = confidence interval

* significant at *p* < 0.05

** significant at *p* < 0.01

For the patient safety and quality issues, management and physicians were significantly more likely than RNs to report high frequency of four of the five issues and the IT staff were more likely to report high frequency of three of the five issues (Table S3). For example, as shown in Table 3, physicians (OR = 2.03), IT staff (OR = 1.72), and management (OR = 1.34) were significantly more likely than RNs to report high frequency of information was not accurate. Physicians (OR = 1.69) and management (OR = 1.27) were also more likely to be dissatisfied with the EHR system than RNs. Note that the regression coefficients by staff position (compared to RNs) were relatively similar across the five patient safety and quality issues (Table S3); thus, we only show the results of one issue (e.g., “High frequency information was not accurate”) in Table 3.

The regression results for the three workflow issues by staff position are available in Supplement Table S4. Compared to RNs, all staff except physicians had significantly higher, favorable mean scores for the workflow item “Enough EHR workstations available when needed” and all staff types had significantly higher scores for “Same information not entered in too many places”. Lastly, compared to RNs, physicians had significantly lower scores for “Not too many alerts or flags in EHR system,” while other clinical, non-clinical, and management staff had significantly higher, more favorable scores.

### Regression results: staff tenure at hospital

In Table 4, we show the multivariable regression results by staff tenure at hospital for the two composite measures, one quality issue, and dissatisfaction with EHR system. Compared to staff with 11 or more years tenure at the hospital, staff with less than 1 year (β = -0.15; *p* < 0.0001) and staff with 1 to 5 years (β = -0.11; *p* < 0.0001) had significantly lower, less favorable mean scores for EHR training. Conversely, for the support and communication composite, staff with tenure of less than one year (β = 0.16; *p* < 0.0001) and 1 to 5 years (β = 0.05; *p* = 0.0387) had significantly higher, more favorable mean scores than the most tenure staff.

For the patient safety and quality issues, in comparison to staff with 11 or more years tenure, staff with less than one year of tenure were significantly less likely to report high frequency of four of the five issues (Table S5). For example, as shown in Table 4, staff with less than 1 year tenure were significantly less likely to report high frequency of information was not accurate (OR = 0.70) than staff with 11 + years tenure. Staff with hospital tenure of 1 to 5 years (OR = 0.79) and 6 to 10 years (OR = 0.85) were significantly less likely to be dissatisfied with the EHR system than the most tenure staff.

Workflow measure results for staff tenure are available in Supplement Table S6. When compared to the 11 or more years of tenure, staff in the categories of less than 1 year and 1 to 5 years had significantly higher and more favorable mean scores of “Same information not entered in too many places” and “Not too many alerts or flags in EHR system” (Table S6).

## Discussion

### EHR-Related issues and workflow

At least 20% of all surveyed staff reported that several EHR-related issues happened at a high frequency of 6 or more times in the past 3 months. Our models found that physicians, management and the IT staff were significantly more likely than RNs to report high frequency of inaccurate information. Furthermore, staff with less than one year of tenure at the hospital reported inaccurate EHR information significantly less often than the highest tenure staff. Similar to how clinicians might notice more data errors that conflict with reality, tenured staff may notice more discrepancies between EHR workflow and the existing workflow, and how the system fails to support the desired care process.

### EHR system training

This composite includes items such as “We are given enough training on how to use the system”, “Training is customized for our work area”, and “We are being adequately trained for when the EHR system is down.” The lowest scores for the EHR training composite were among the pharmacists and the physicians. These results reveal that more research is needed to determine how to improve and better customize EHR training for pharmacists and physicians. The highest scores were among the IT staff, mostly likely due to the fact that these systems are implemented and managed by the IT staff and because the IT staff has less knowledge of real-world clinical needs.

As EHR and Health IT applications evolve and are used more across the continuum of care, health care organizations should work to increase the health IT literacy of pharmacists and clinicians, so they can assist in the design and feedback of user friendly EHR systems and improve the diagnostic process and quality and coordination of patient care [36, 37]. Optimizing health IT literacy pertains to healthcare staff of all tenure level, even new and less tenured staff, to avoid setbacks and errors.

Interestingly, new staff to the hospital had the lowest scores for EHR training. New staff, that may include young nurses, medical students & residents, are generally a young population, with more familiarity with technology, and, thus, they may have less patience with basic computer skill and user interface (UI) training. Among other staff types, those with less tenure most likely do more data entry and need better training in that regard. In contrast, staff with the longest tenure had the best perceptions of EHR training. Staff with more tenure may have more favorable perceptions about EHR training and didactics than those with less tenure because they have been actively learning the hospital’s EHR system for many years, are more familiar with the system, and/or are less interested in training. When they do receive training, the more tenured staff may understand the training better than less tenured staff due to having more experience with the hospital’s EHR system’s features.

### EHR system support & communication

Pharmacists, physicians, and RNs had the lowest scores for the support & communication composite, which included questions about problems being resolved in a timely manner, if input for improvement is requested, and being made aware of issues that could lead to more errors. These findings suggest that hospitals need to better engage the end-user clinicians for their feedback and recommendations regarding EHR processes. This is important because the more-tenured staff most likely have a deeper understanding of the system’s workflow issues and threats to patient safety.

Across four categories of tenure, the most tenured staff group had significantly lower (unfavorable) scores for support & communication than the staff groups with less than 5 years at the hospital. Again, this is concerning because, in comparison to new staff, the more tenured may be more reliant on technical support and peer support than they are on training.

Thus, hospitals should provide feedback to all staff on EHR-related patient safety issues and seek input from the most tenured staff on ways to improve a hospital’s EHR system, but without the threat of retribution. For example, innovative ideas to improve hospital EHR training, support, processes, and organizational learning could be achieved through anonymous interviews with physicians [38] and the implementation of an interdisciplinary patient safety learning laboratory that fosters innovation [39].

### EHR system dissatisfaction

Staff with 11 or more years at the hospitals were significantly more likely to be dissatisfied with the EHR system in comparison to staff with 1–10 years at the hospital. This may be a behavioral issue related to resistance to change and/or a reflection of the more tenured staff having to address persistent issues leading to dissatisfaction. This is revealing, as more tenured staff have the most experience and knowledge of the hospital’s processes and are most often in leadership and mentoring roles.

Physicians, RNs, and management were the staff types most likely to be dissatisfied with the EHR system. Surprisingly, management displayed a significant higher likelihood of EHR system dissatisfaction compared to RNs. While we can understand the dissatisfaction of the EHR system by physicians and RNs (as displayed by low scores for support and communication), future studies should explore the factors associated with management’s dissatisfaction with the EHR system.

### Implications

Our findings lead to recommendations for improved EHR training for pharmacists and physicians which include role-specific customization of EHR training modules to address the lower satisfaction scores identified among these two types of healthcare professionals.

Vendors and EHR developers must engage clinician feedback during product design and implementation to meet clinicians’ needs and to make the EHR system more clinically intuitive and user-friendly [40]. Hospitals should encourage all staff who interact with patients to speak up and acknowledge workflow changes that threaten patient safety and/or reduce efficiency [41]. For example, a study that looked at EHR-related physician distress found that physicians would not share problems due to lack of confidence that the problems would be addressed and possible retribution [38]. Researchers have also found that nurses’ ability to adopt work-arounds to provide patient care as they experience EHR-related unintended consequences offers new perspectives and innovative ideas for improving the EHR [42].

Thus, hospitals should increase the solicitation of input from clinicians on how to improve hospital’s EHR system for safer care, better coordinated care, and to decrease diagnostic error [43, 44]. The input and feedback from clinicians, pharmacists, and RNs are especially important when an organizational “stressor” threatens EHR system reliability, diagnostic capability, and the process of care. Examples of stressors are an EHR transition such as a new or upgraded system implementation; the implementation of an evidence-based, quality improvement intervention; or an external event such as the COVID19 pandemic [45, 46].

Lastly, we recommend that hospitals use both Hospital SOPS and the Health IT item set before and after QI interventions and EHR process change to measure causal inference and to evaluate effectiveness, perceptions of safety, and dissatisfaction. Furthermore, to adequately measure and compare patient safety culture across staff type, hospitals should consider strategies to enhance physician participation with the survey.

### Limitations

The study had several limitations. First, the study may include bias due to the voluntary nature of the 2017 pilot study of the SOPS Health IT item set. This may have resulted in disproportionate inclusion of hospitals in our sample with patient safety better than the national norm. However, because the average site response rate was 46% (range: 20–74%), there may be individual-level response bias as those who felt compelled to participate may have done so because they perceived lower safety. Furthermore, the relatively small sample size of the physician group respondents (*n* = 524) may have resulted in false negative significant differences in the perceptions of the EHR system in comparison to RNs, and the physician group’s responses may not be generalizable to the population of physicians working in hospitals. Second, there may be a recall bias to events such as EHR training, especially among staff with more tenure at the hospital. We recognize that a new EHR system would include more recent trainings to everyone. But unfortunately, our data did not include how long a hospital had their current EHR system in question. Lastly, the dependent variables regarding EHR safety may not be truly independent from a staff member’s overall dislike of EHRs, in general.

## Conclusions

The SOPS Health IT Patient Safety Supplemental Item Set for hospitals is a valuable tool to assess provider and staff perceptions of EHR satisfaction, training, support & communication, and patient safety issues. We found that management, the IT staff, and the physicians are the most aware among staff groups of the problems associated with their hospital’s EHR system and implementation. Our results suggest that hospitals and EHR developers should increase the solicitation of input and feedback from clinicians and more tenured staff on how to improve the safety and usability of a hospitals’ EHR system. The findings also lead to recommendations for immediate review of EHR training programs to address the disparities in training satisfaction across different staff groups.

### Electronic supplementary material

Below is the link to the electronic supplementary material.

Supplementary Material 1

## Data Availability

De-identified SOPS Hospital Survey 1.0 data are available upon request for research purposes from the Surveys on Patient Safety Culture^®^ (SOPS^®^) Hospital Database.

## Abbreviations

AHRQ: Agency for Healthcare Research and Quality
ANOVA: Analysis of variance
EHR: Electronic health record
IRB: Institutional review board
IT: Information technology
OR: Odds ratio
RN: Registered nurse
SAS: Previously “Statistical Analysis System”
SOPS^®^: Surveys on Patient Safety Culture^®^
