# Supplementary material for: Perceptions of hospital electronic health record (EHR) training, support, and patient safety by staff position and tenure
Source: BMC Health Serv Res. 2024 Aug 20;24:955. doi: 10.1186/s12913-024-11322-3 (PMC11337607; doi:10.1186/s12913-024-11322-3)
Supplement: Supplementary file 1 — Supplementary Material 1 [file 12913_2024_11322_MOESM1_ESM.docx]

**Table S1. AHRQ Hospital SOPS® Health IT Supplemental Item Set**

| **Section** | **Question(s)** | **Response Options** | **# of Outcome Measures / Score** |
| --- | --- | --- | --- |
| 1. EHR Patient Safety and Quality Issues | In the past 3 months, how many times did you discover the following issues with the EHR system in your hospital?   1. Information was not complete 2. Information was not accurate 3. Important information was hard to find 4. Information was entered into the wrong patient health record 5. Incorrect information was copied and pasted | 1. = none 2. = 1-5 times 3. = 6-10 times 4. = 11-20 times 5. = 21-50 times 6. = more than 50 times   *N/A or DK (set to missing)* | Five measures  Dichotomized response:  1 = High frequency (6 or more times)  0 = Not high frequency (none or 1-5 times) |
| B. EHR System Training (Composite Measure) | How much do you agree or disagree with the following statements?   1. We are given enough training on how to use our EHR system 2. Training on our EHR system is customized for our work area 3. We are adequately trained on what to do when our EHR system is down | 1 = strongly disagree  2 = disagree  3 = neither agree nor disagree  4 = agree  5 = strongly agree  *N/A or DK (set to missing)* | One measure  Composite measure **mean score^a^** of the 3 items ranging from 1-5 (higher is better) |
| C. EHR and Workflow/Work Process | How much do you agree or disagree with the following statements?   1. There are enough EHR workstations available when we need them 2. Our EHR system requires that we enter the same information in too many places (R) 3. There are too many alerts or flags in our EHR system (R) | 1 = strongly disagree  2 = disagree  3 = neither agree nor disagree  4 = agree  5 = strongly agree  *N/A or DK (set to missing)* | Three measures  Score ranging from 1-5 (higher is better) |
| D. EHR System Support and Communication (Composite Measure) | How much do you agree or disagree with the following statements?  1. Problems with our EHR system are resolved in a timely manner  2. We are asked for input on ways to improve our EHR system  3. We are made aware of issues with our EHR system that could lead to more errors | 1 = strongly disagree  2 = disagree  3 = neither agree nor disagree  4 = agree  5 = strongly agree  *N/A or DK (set to missing)* | One measure  Composite measure **mean score^a^** of the 3 items ranging from 1-5 (higher is better) |
| E. Overall EHR System Rating | How satisfied or dissatisfied are you with your hospital’s EHR system? | 1 = very dissatisfied  2 = dissatisfied  3 = neither satisfied nor  dissatisfied  4 = satisfied  5 = very satisfied | One measure  Dichotomized response:  1 = Dissatisfied (1 or 2)  0 = Not dissatisfied (3-5) |

Note: (R) indicates a negatively worded item and the value is reversed for scoring. For example, in calculating the scores, strongly disagree will receive a 5 instead of a 1. N/A = not applicable or does not apply; DK = don’t know.

^a^ Composite measure means were calculated only when all 3 items were answered with a valid 1-5 response.

**Table S2. Staff Position Group**

| **Staff Position Group** | **Self-Reported Staff Positions** |
| --- | --- |
| Administration, Management | Manager, Department Manager, Administrator, Director, Senior Leader, Executive, C-Suite |
| Information Technology | Health Information Technology (Health IT), Clinical Informatics, Health Information Services |
| Medical Assistant, Other Clinical | Licensed Vocational Nurse (LVN), Licensed Practical Nurse (LPN), Advanced Practice Nurse (NP, CRNA, CNS, CNM), Patient Care Aide, Nursing Assistant, Physician Assistant, Dietician, Therapist (Physical, Respiratory, Occupational, or Speech), Psychologist, Medical Assistant |
| Non-Clinical, Office, Social Work | Unit Clerk, Secretary, Receptionist, Office Staff, Finance, Billing, Analyst, Registrar, Eligibility Specialist, Social work (SW), Case management (CM) |
| Pharmacist, Technician | Pharmacist, Technologist, Technician (laboratory, radiology, pharmacy, surgical, electrocardiogram) |
| Physician, Resident | Physician, Attending, Hospitalist, Surgeon, Resident, Intern |
| Registered Nurse | Registered Nurse (RN) |

**Table S3. Logistic Regression Results: High Frequency of Patient Safety and Quality Issues by Staff Position**

|  | Information Not Complete | | Information Not Accurate | | Important Information Hard to Find | | Information Entered into Wrong EHR | | Incorrect Information Copied and Pasted | |
| --- | --- | --- | --- | --- | --- | --- | --- | --- | --- | --- |
|  | OR | 95% CI | OR | 95% CI | OR | 95% CI | OR | 95% CI | OR | 95% CI |
| Position Type (ref = RN) |  |  |  |  |  |  |  |  |  |  |
| Administration, Management | **1.36*** | (1.11,1.66) | **1.34*** | (1.07,1.68) | **1.60*** | (1.33,1.94) | 1.54 | (0.95,2.51) | **1.55*** | (1.19,2.02) |
| Information Technology | **1.65*** | (1.26,2.15) | **1.72*** | (1.29,2.28) | 0.98 | (0.75,1.28) | **5.50*** | (3.66,8.27) | 1.11 | (0.74,1.67) |
| Medical Assistant, Other Clinical | **0.68*** | (0.58,0.79) | **0.81*** | (0.68,0.96) | **0.72*** | (0.63,0.84) | 1.07 | (0.73,1.56) | 0.84 | (0.68,1.04) |
| Non-Clinical, Office, Social Worker | **0.64*** | (0.54,0.77) | 0.85 | (0.70,1.04) | **0.45*** | (0.37,0.54) | 1.19 | (0.78,1.80) | **0.41*** | (0.29,0.56) |
| Pharmacist, Technician | 0.87 | (0.72,1.05) | 0.91 | (0.74,1.13) | **0.74*** | (0.61,0.88) | 0.85 | (0.50,1.43) | **0.42*** | (0.30,0.60) |
| Physician, Resident | **1.74*** | (1.41,2.13) | **2.03*** | (1.64,2.52) | **1.89*** | (1.55,2.30) | 1.61 | (0.99,2.63) | **2.20*** | (1.72,2.82) |
| Bed size (ref = 100 – 299) |  |  |  |  |  |  |  |  |  |  |
| 50-99 | 0.94 | (0.97,1.24) | 0.93 | (0.77,1.12) | 0.97 | (0.82,1.13) | 0.85 | (0.55,1.29) | 0.86 | (0.67,1.12) |
| 300 or more | 1.10 | (0.79,1.11) | 1.08 | (0.94,1.23) | 1.09 | (0.97,1.22) | 1.11 | (0.83,1.48) | **1.29*** | (1.10,1.52) |
| Ownership (ref = Nongovt nonprofit) |  |  |  |  |  |  |  |  |  |  |
| Government nonfederal | 1.03 | (0.83,1.26) | 0.96 | (0.77,1.21) | 0.97 | (0.80,1.19) | **1.55*** | (1.02,2.36) | **0.50*** | (0.35,0.72) |
| For profit | 1.06 | (0.77,1.46) | 0.85 | (0.58,1.23) | 0.81 | (0.59,1.12) | 1.55 | (0.76,3.18) | **0.46*** | (0.24,0.87) |

Note: n = 8880; odds ratio > 1 (unfavorable) indicates greater likelihood of high frequency issues in comparison to the reference group.

Ref = reference; RN = Registered Nurse; Nongovt = nongovernment; OR = odds ratio; CI = confidence interval

* significant at p < 0.05

**Table S4. Regression Results: Workflow Issues by Staff Position**

|  | Enough EHR Workstations Available When Needed | | Same Information Not Entered in Too Many Places | | Not Too Many Alerts or Flags in EHR System | |
| --- | --- | --- | --- | --- | --- | --- |
|  | Coefficient  Estimate | Pr > \|t\| | Coefficient  Estimate | Pr > \|t\| | Coefficient  Estimate | Pr > \|t\| |
| Position Type (ref = RN) |  |  |  |  |  |  |
| Administration, Management | **0.47**** | <.0001 | **0.21**** | <.0001 | **0.11*** | 0.0140 |
| Information Technology | **0.49**** | <.0001 | **0.66**** | <.0001 | 0.01 | 0.8549 |
| Medical Assistant, Other Clinical | **0.07*** | 0.0265 | **0.38**** | <.0001 | **0.11**** | 0.0002 |
| Non-Clinical, Office, Social Worker | **0.27**** | <.0001 | **0.51**** | <.0001 | **0.15**** | <.0001 |
| Pharmacist, Technician | **0.17**** | <.0001 | **0.54**** | <.0001 | -0.02 | 0.5106 |
| Physician, Resident | 0.03 | 0.5138 | **0.19**** | 0.0003 | **-0.39**** | <.0001 |
| Bed size (ref = 100 – 299) |  |  |  |  |  |  |
| 50-99 | **0.52**** | 0.0037 | **-0.42*** | 0.0182 | **-0.33*** | 0.0496 |
| 300 or more | -0.05 | 0.5866 | -0.08 | 0.3729 | **-0.20*** | 0.0357 |
| Ownership (ref = Nongovt nonprofit) |  |  |  |  |  |  |
| Government nonfederal | -0.37 | 0.0701 | 0.28 | 0.1661 | 0.26 | 0.1733 |
| For profit | **-0.71**** | 0.0013 | 0.19 | 0.3716 | 0.11 | 0.5798 |

Note: n = 8880; a positive regression coefficient indicates better mean score on the item in comparison to the reference group.

A negative coefficient indicates a less favorable mean score.

Ref = reference; RN = Registered Nurse; Nongovt = nongovernment; Pr = probability

* significant at p < 0.05

** significant at p < 0.01

**Table S5. Logistic Regression Results: High Frequency of Patient Safety and Quality Issues by Hospital Tenure**

|  | Information Not Complete | | Information Not Accurate | | Important Information Hard to Find | | Information Entered into Wrong EHR | | Incorrect Information Copied and Pasted | |
| --- | --- | --- | --- | --- | --- | --- | --- | --- | --- | --- |
|  | OR | 95% CI | OR | 95% CI | OR | 95% CI | OR | 95% CI | OR | 95% CI |
| Hospital Tenure (ref = 11 or more years) |  |  |  |  |  |  |  |  |  |  |
| Less than 1 year | **0.64*** | (0.52,0.79) | **0.70*** | (0.56,0.88) | **0.83*** | (0.69,0.99) | 0.94 | (0.57,1.57) | **0.55*** | (0.40,0.75) |
| 1 to 5 years | **0.82*** | (0.72,0.93) | 0.96 | (0.84,1.10) | 0.90 | (0.80,1.02) | 1.11 | (0.80,1.54) | 0.92 | (0.77,1.10) |
| 6 to 10 years | 0.93 | (0.80,1.09) | 0.93 | (0.78,1.10) | 0.95 | (0.82,1.10) | 1.30 | (0.89,1.89) | 1.20 | (0.97,1.48) |
| Bed size (ref = 100 – 299) |  |  |  |  |  |  |  |  |  |  |
| 50-99 | 0.91 | (0.76,1.08) | 0.90 | (0.74,1.09) | 0.98 | (0.83,1.15) | 0.71 | (0.44,1.15) | 0.83 | (0.64,1.08) |
| 300 or more | **1.14*** | (1.01,1.29) | 1.13 | (0.99,1.29) | **1.16*** | (1.04,1.30) | 1.06 | (0.78,1.44) | **1.40*** | (1.19,1.65) |
| Ownership (ref = Nongovt nonprofit) |  |  |  |  |  |  |  |  |  |  |
| Government nonfederal | 0.99 | (0.80,1.23) | 0.92 | (0.73,1.17) | 0.94 | (0.76,1.15) | 1.45 | (0.89,2.34) | **0.44*** | (0.30,0.65) |
| For profit | 1.04 | (0.74,1.45) | 0.85 | (0.58,1.25) | 0.75 | (0.54,1.04) | 1.70 | (0.76,3.77) | **0.40*** | (0.20,0.81) |

Note: n = 8548 (excluding IT staff); odds ratio > 1 (unfavorable) indicates greater likelihood of high frequency issues in comparison to the reference group.

Ref = reference; Nongovt = nongovernment; OR = odds ratio; CI = confidence interval

* significant at p < 0.05

**Table S6. Regression Results: Workflow Issues by Hospital Tenure**

|  | Enough EHR Workstations Available When Needed | | Same Information Not Entered in Too Many Places | | Not Too Many Alerts or Flags in EHR System | |
| --- | --- | --- | --- | --- | --- | --- |
|  | Coefficient  Estimate | Pr > \|t\| | Coefficient  Estimate | Pr > \|t\| | Coefficient  Estimate | Pr > \|t\| |
| Hospital Tenure (ref = 11 or more years) |  |  |  |  |  |  |
| Less than 1 year | 0.04 | 0.3523 | **0.15**** | 0.0007 | **0.16**** | <.0001 |
| 1 to 5 years | -0.04 | 0.2317 | **0.10**** | 0.0010 | **0.08**** | 0.0014 |
| 6 to 10 years | **-0.10**** | 0.0064 | <0.01 | 0.9629 | -0.05 | 0.0838 |
| Bed size (ref = 100 – 299) |  |  |  |  |  |  |
| 50-99 | **0.49**** | 0.0076 | **-0.46*** | 0.0121 | **-0.34*** | 0.0339 |
| 300 or more | -0.06 | 0.4806 | -0.09 | 0.3237 | **-0.20*** | 0.0110 |
| Ownership (ref = Nongovt nonprofit) |  |  |  |  |  |  |
| Government nonfederal | -0.40 | 0.0634 | 0.23 | 0.2723 | 0.23 | 0.2201 |
| For profit | **-0.71**** | 0.0019 | 0.21 | 0.3513 | 0.10 | 0.6126 |

Note: n = 8548 (excluding IT staff); a positive regression coefficient indicates better mean score on the item in comparison to the reference group.

A negative coefficient indicates a less favorable mean score.

Ref = reference; Nongovt = nongovernment; Pr = probability

* significant at p < 0.05

** significant at p < 0.01
